# Prevalence and Clinical Associations of Germline DDR Variants in Prostate Cancer: Real-World Evidence from a 122-Patient Turkish Cohort

**DOI:** 10.3390/genes17010023

**Published:** 2025-12-26

**Authors:** Seval Akay, Taha Resid Ozdemir, Ozge Ozer Kaya, Mustafa Degirmenci, Olcun Umit Unal

**Affiliations:** 1 Medical Oncology Department, Izmir City Hospital, Izmir 35400, Türkiye; degirmencigil@gmail.com (M.D.); drolcun@hotmail.com (O.U.U.); 2 Medical Genetics Department, Izmir City Hospital, Izmir 35400, Türkiye; dr.tahaoz@gmail.com (T.R.O.); drozgeozer@gmail.com (O.O.K.)

**Keywords:** prostate cancer, germline variants, DNA damage repair, homologous recombination, CHEK2, BRCA2, variants of uncertain significance

## Abstract

Background: Germline alterations in DNA damage repair (DDR) genes represent a clinically important subset of prostate cancer (PCa), but real-world data from Middle Eastern and Turkish populations remain limited. We evaluated the prevalence and clinicopathologic associations of germline DDR variants in a single-center Turkish cohort. Methods: We retrospectively analyzed 122 men with histologically confirmed PCa who underwent germline multigene panel testing. Variants were classified according to ACMG/ClinVar criteria. Patients were grouped as pathogenic/likely pathogenic (P/LP), variants of uncertain significance (VUS), or variant-negative. Patients were grouped as variant-positive (P/LP or VUS/uncategorized) or clinically actionable variant–negative (benign/likely benign or no variant detected). Group comparisons used *t*-tests, chi-square or Fisher’s exact tests as appropriate. Results: The median age at diagnosis was 65.2 years (mean 64.6 ± 8.78). Overall, 37 patients (30.3%) carried at least one germline variant, including 12 (9.8%) with P/LP alterations and 24 (19.7%) with VUS; one patient (0.8%) harbored an uncategorized variant. The most frequently affected genes were CHEK2 (*n* = 8), BRCA1 (*n* = 6), BRCA2 (*n* = 6), ATM (*n* = 5), and APC (*n* = 4). Variant-positive status increased from 10.8% in ISUP 1–2 to 21.6% in ISUP 3 and 76.0% in ISUP 4–5, although this trend was not statistically significant (*p* = 0.391). Mean age at diagnosis and the prevalence of metastatic disease did not differ between variant-positive and clinically actionable variant–negative patients (64.2 vs. 65.7 years, *p* = 0.390; 66.7% vs. 64.6%, *p* = 0.842). Truncating DDR variants (RAD50, BRCA2, MSH3, NBN, CHEK2, ATM) occurred predominantly in ISUP 4–5 tumors. Conclusions: Germline DDR alterations—most notably in BRCA2, CHEK2, and ATM—were present in a substantial subset of Turkish men with PCa and showed a non-significant trend toward clustering in higher-grade disease. The high prevalence of VUS reflects limited genomic annotation in under-represented populations and underscores the need for longitudinal reinterpretation. These data support the clinical value of incorporating germline DDR testing into risk assessment and familial counseling, while larger cohorts integrating somatic profiling are needed to refine genotype–phenotype associations.

## 1. Introduction

Cancer treatment has undergone a major conceptual shift over recent decades, evolving from predominantly empirical, histology-driven approaches toward increasingly molecularly informed and precision-based strategies. Early therapeutic paradigms relied largely on surgery, radiotherapy, and non-selective cytotoxic chemotherapy, with treatment decisions guided primarily by tumor stage and anatomical extent. While these approaches achieved meaningful survival improvements, they were frequently limited by substantial toxicity and marked inter-patient variability in treatment response. Advances in cancer biology and genomics have since reshaped this landscape, enabling the identification of actionable molecular alterations and the integration of targeted therapies, immunotherapy, and biomarker-guided treatment selection into routine clinical practice. As comprehensively reviewed by Sonkin and Thomas, modern oncology increasingly emphasizes the convergence of clinical phenotyping with genomic profiling to refine prognostication, personalize therapy, and better understand mechanisms of resistance and disease progression [1].

Within this evolving framework, prostate cancer (PCa) represents a biologically heterogeneous disease characterized by a diverse spectrum of molecular alterations that collectively influence tumor initiation, progression, and clinical aggressiveness. Large-scale sequencing studies have demonstrated recurrent disruptions in androgen receptor (AR) signaling, DNA damage repair (DDR) pathways, PI3K/AKT signaling, and frequent loss of critical tumor suppressors such as TP53, PTEN, and RB1, underscoring the multiplicity of prognostic and potentially actionable molecular events in this malignancy [2,3]. Among these, inherited germline alterations in DDR genes—particularly those involved in homologous recombination repair (HRR), including BRCA2, BRCA1, ATM, CHEK2, and PALB2—have been associated with distinct molecular and clinical features in subsets of prostate cancer. Reflecting this, contemporary NCCN guidelines endorse germline multigene testing for HRR defects and assessment for mismatch repair deficiency (dMMR) or microsatellite instability (MSI-H), particularly in patients with regional or metastatic disease [4]. Notably, recent population-based evaluations show that germline HRR alterations occur in a meaningful proportion of men with advanced PCa and carry important implications for genetic counseling and multidisciplinary management [5]. Beyond HRR-related germline susceptibility, additional emerging molecular subsets—including dMMR/MSI-H tumors and biallelic CDK12 inactivation—represent mechanistically distinct categories with unique oncogenic trajectories [6,7].

In advanced and metastatic prostate cancer, defects in DDR pathways have been closely linked to increased chromosomal instability, accumulation of genomic rearrangements, and aggressive tumor behavior. Both germline and somatic alterations in key DDR genes have been linked to genomic instability and aggressive biological behavior in advanced disease, although the clinical impact of individual germline alterations remains incompletely defined [3,4,5].

Despite the growing body of evidence linking germline DDR alterations to disease aggressiveness and treatment responsiveness, data from Middle Eastern and Turkish populations remain limited. In one of the few available Turkish series, Manguoğlu et al. reported germline BRCA1/2 mutations in high-risk patients with breast, ovarian, and PCa, highlighting the genetic heterogeneity of this population and the need for more comprehensive, PCa-focused genomic profiling [8]. Such gaps in population-specific data hinder population-specific understanding of germline variant distributions and their clinicopathological context. Moreover, while prior clinicogenetic association studies have established the importance of systematically linking germline variants with clinicopathological features, comparable structured analyses remain scarce in real-world PCa cohorts from this region. Therefore, this study aimed to describe the prevalence and clinicopathological distribution of germline DDR variants in a real-world cohort of Turkish men with prostate cancer, with particular attention to tumor grade, stage, metastatic status, and the distribution of variants of uncertain significance.

## 2. Materials and Methods

### 2.1. Clinical Cohort

This retrospective cohort study included patients diagnosed with prostate adenocarcinoma who underwent germline genetic testing and clinical staging at our institution. Genetic testing was performed as part of routine clinical diagnostic evaluation, independent of this study. Clinical records, pathology reports, and genomic profiles were reviewed. Patients were eligible if both date of birth and date of diagnosis were available to calculate age at diagnosis. Patients without clinically relevant variants (benign, likely benign, or no variant detected) were retained for comparison as the variant-negative group.

The inclusion criteria consisted of histologically confirmed prostate adenocarcinoma from biopsy or prostatectomy specimens; clinical stage T1–T4 at diagnosis, availability of sufficient peripheral blood-derived DNA for germline testing; and documented presence or absence of secondary malignancy when applicable.

Gleason score and ISUP Grade Group were abstracted from pathology reports and categorized as follows:

Grade Group 1 = Gleason ≤ 6

Grade Group 2 = Gleason 3 + 4

Grade Group 3 = Gleason 4 + 3

Grade Group 4 = Gleason 8

Grade Group 5 = Gleason 9–10

Staging was performed based on the TNM criteria according to *AJCC* 8th edition; missing TNM data were retained in the database and treated as missing-at-random.

Ethical approval for this retrospective study was obtained from the Institutional Review Board of Izmir Tepecik Training and Research Hospital (Approval No: 2022/02-10). The requirement for informed consent was waived by the ethics committee because the study involved de-identified retrospective data and posed no additional risk to participants. All procedures were conducted in accordance with the Declaration of Helsinki.

### 2.2. Genomic DNA Isolation and Sequencing

Genomic DNA was isolated from all study participants using the MagPurix Blood DNA Extraction Kit (Zinexts Life Science Corp., New Taipei City, Taiwan) in accordance with the manufacturer’s protocol. Germline next-generation sequencing (NGS) was subsequently performed using a comprehensive hereditary cancer panel targeting 42 genes implicated in DNA repair pathways and hereditary tumor predisposition (the full list of analyzed genes is provided in Figure S1).

### 2.3. Bioinformatic Processing and Variant Filtering

Raw sequence data were analyzed using SEQ software V8.16.0 (Genomize, İstanbul, Turkey) with alignment to the GRCh38 human reference genome. All targeted regions achieved a minimum sequencing depth of 50× before variant filtration. A multi-step filtering strategy was applied:

1. Variants classified as Benign (B) or Likely Benign (LB) in all ClinVar submissions were excluded.

2. Variants with an allele frequency greater than 5% in population databases (1000 Genomes, ExAC, ESP) were removed.

3. Variants located within coding regions or relevant intronic sequences were retained for interpretation.

### 2.4. Variant Interpretation and Validation

All remaining variants were evaluated and classified in accordance with the American College of Medical Genetics and Genomics (ACMG) and the Association for Molecular Pathology (AMP) guidelines for sequence variant interpretation [9].

All variants deemed pathogenic or likely pathogenic through NGS were confirmed using Sanger sequencing. Validation was performed on an ABI PRISM 3500 DNA Analyzer (Applied Biosystems, Foster City, CA, USA) following standard laboratory procedures.

To ensure consistency in the interpretation of variants, all genetic results, including variant pathogenicity classification, were analyzed by a single experienced medical geneticist. variant interpretation was performed without knowledge of clinical information, such as PSA values, Gleason score, ISUP grade, and TNM stage, to avoid any potential bias.

All reported germline variants were identified in the heterozygous state; no homozygous or compound heterozygous pathogenic variants were detected.

### 2.5. Variant Categorization

For analytical clarity, patients were grouped as:

- pathogenic/likely pathogenic carriers (clinically actionable)
- VUS carriers
- variant-negative

Variants interpreted as benign or likely benign were combined into one category with patients having no detectable alterations. Evidence used for classification included variant type (e.g., truncating, frameshift, nonsense, missense), allele frequency in population databases, ClinVar submissions, previously reported disease associations, and predicted impact on protein function. Variants classified as pathogenic or likely pathogenic were confirmed by Sanger sequencing. Final variant classification was performed by an experienced medical geneticist following ACMG/AMP criteria.

### 2.6. Statistical Analysis

Continuous variables were assessed with the Shapiro–Wilk test. Normally distributed variables were compared using Student’s *t*-test, whereas non-normally distributed variables were analyzed using the Mann–Whitney U test. Categorical comparisons were made by chi-square or Fisher’s exact test. A *p*-value < 0.05 was considered statistically significant. Statistical analyses were carried out with SPSS (IBM SPSS Statistics, Version 25.0).

## 3. Results

A total of 122 patients were analyzed. The median age at diagnosis was 65.2 years (mean 64.6 ± 8.78), with 25th, 50th, and 75th percentiles of 59.1, 65.2, and 70.7 years, respectively. Of the cohort, 85 patients (69.7%) were classified as clinically actionable variant–negative, whereas 37 patients (30.3%) carried at least one germline variant. Among these, 12 (9.8%) harbored pathogenic or likely pathogenic (P/LP) alterations, 24 (19.7%) carried variants of uncertain significance (VUS), and one patient (0.8%) had an uncategorized variant. Individual-level clinical and germline genetic characteristics of all patients are provided in Supplementary Table S1.

The ISUP Grade Group distribution was: Grade Group 1 (*n* = 9, 7.4%), Grade Group 2 (*n* = 13, 10.7%), Grade Group 3 (*n* = 24, 19.7%), Grade Group 4 (*n* = 27, 22.1%), and Grade Group 5 (*n* = 49, 40.2%). A summarized overview of germline variant distribution across ISUP Grade Groups is presented in Table 1. Variant-carrying status was numerically higher in intermediate- and high-grade disease; although this association did not reach statistical significance (*p* = 0.259) and should therefore be interpreted as descriptive and exploratory.

Age was normally distributed across groups (Shapiro–Wilk *p* = 0.226), and mean age at diagnosis was similar between clinically actionable variant–negative and variant-positive patients (64.2 vs. 65.7 years; *p* = 0.390). The proportion of metastatic disease at diagnosis was also comparable between these groups (66.7% vs. 64.6%; *p* = 0.842).

**Figure 1:**
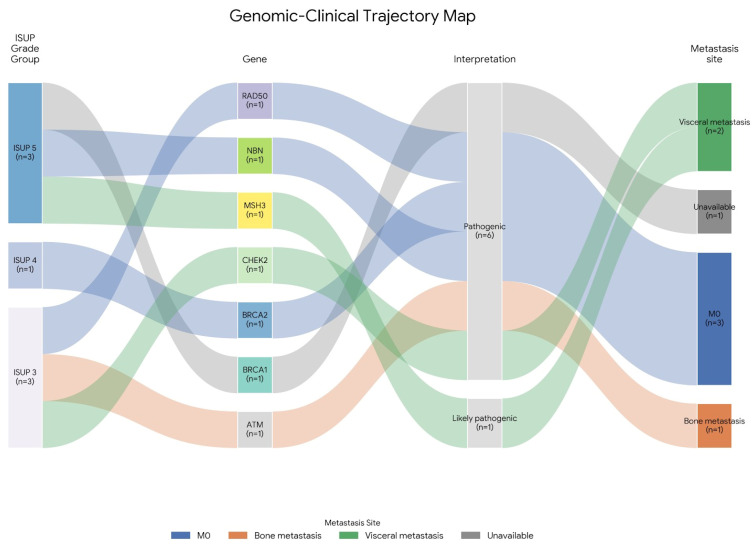
Genomic–clinical trajectory map of pathogenic germline truncating DDR variants. Alluvial diagram illustrating patient-level transitions from ISUP Grade Group to the affected DDR gene, variant interpretation, and metastatic status. Flow widths are proportional to the number of patients (n). Variant classification (pathogenic or likely pathogenic) was based on standard clinical interpretation criteria. Metastatic status is categorized as M0 (no metastasis), bone metastasis, visceral metastasis, or unavailable. The figure demonstrates the heterogeneity of high-grade prostate cancer in terms of germline DDR alterations and associated metastatic patterns. The full name of genetic variations were as follows: BRCA1(NM_007294.4): c.1082C>G(p.Ser361Ter), RAD50(NM_005732.4): c.326_329del(p.Thr109fs), BRCA2(NM_000059.4): c.3751dup(p.Thr1251fs), NBN(NM_002485.5): c.657_661del(p.Lys219fs), ATM(NM_000051.4): c.3576G>A (p.Lys1192=), CHEK2(NM_007194.4): c.1100del(p.Thr367fs), and MSH3(NM_002439.5): c.3046_3050delinsTCA(p.Glu1016SerfsTer20).

Among variant-positive patients with metastatic disease, 75% presented with nodal, skeletal, or visceral metastases, reflecting the typical distribution of metastatic involvement observed in advanced prostate cancer. The most frequently altered genes were CHEK2 (*n* = 8), BRCA1 (*n* = 6), BRCA2 (*n* = 6), ATM (*n* = 5), and APC (*n* = 4), with additional variants detected in MSH6, MSH3, and NBN. A total of 11 truncating or clearly deleterious variants were identified, including frameshift alterations in RAD50, BRCA2, MSH3, NBN, CHEK2, and ATM. These loss-of-function variants were more frequently observed in patients with ISUP Grade Group 4–5 tumors and are summarized in Figure 1.

Pathogenic variants in BRCA2, CHEK2, and ATM were observed more frequently in higher-grade tumors, representing a non-significant directional trend. However, no association was identified between pathogenic variant status and pathological stage or metastatic presentation. In contrast, variants in STK11, PMS2, MUTYH, and RB1 were primarily classified as VUS and did not demonstrate an apparent relationship with tumor aggressiveness.

Three patients were diagnosed with secondary malignancies (melanoma, bladder carcinoma, and pulmonary carcinoma). Each case carried VUS in genes related to DNA repair (POLD1, BARD1, or BRIP1); however, given the uncertain classification of these variants, no direct inference regarding hereditary cancer predisposition can be made.

## 4. Discussion

In this real-world cohort of 122 Turkish men with PCa, we provide an updated characterization of the germline DDR landscape using a comprehensive 42-gene hereditary cancer panel. Three key observations emerged from our analysis: (i) the prevalence of clinically actionable DDR alterations was similar to that documented in contemporary international cohorts, (ii) carriers of P/LP variants showed a descriptive enrichment in higher ISUP grade tumors, without reaching statistical significance and (iii) VUS constituted a substantial proportion of the detected variants.

Our findings are consistent with several recent genomic analyses demonstrating that 8–15% of men with PCa cases carry P/LP germline variants in DDR-related genes, including the most commonly altered, BRCA2, followed by ATM, CHEK2 and other HRR-pathway genes [10,11,12]. Large population-based studies have also established an increased risk for BRCA2 carriers of early-onset disease, higher tumor grade and metastatic presentation, whereas other DDR genes exhibit more heterogeneous and gene-specific penetrance [13,14,15]. Consistent with prior reports suggesting an association between DDR alterations and aggressive disease features, BRCA2, ATM and CHEK2 carriers in our cohort were more frequently observed among ISUP 4–5 tumors; however, this trend did not reach statistical significance (*p* = 0.259), suggesting that, while a directional trend toward more aggressive disease biology may be present in P/LP carriers, larger cohorts would be required to confirm this.

A notable finding of our study is that age at diagnosis and rates of metastatic disease were similar between variant-negative and variant-positive individuals (*p* = 0.390 and *p* = 0.842, respectively), suggesting that germline DDR variants alone may not uniformly dictate metastatic potential. This emphasizes the multifactorial nature of metastatic progression in PCa, in which somatic evolution, genomic instability, tumor microenvironment interactions and treatment timing may interact with the germline background [16,17,18,19]. The integration of somatic sequencing—absent in our study—will be essential to fully delineate how germline DDR alterations interface with tumor evolution and therapeutic responsiveness.

Our cohort also demonstrated a substantial VUS burden (19.7%), comparable to recent genomic reports from genetically under-represented patient populations [20,21]. Consistent with ACMG-guided re-evaluation studies, approximately 75–90% of these VUS are ultimately downgraded to benign or likely benign over time [22,23]. Therefore, VUS in the present study should be interpreted cautiously, and the present findings should not be used in isolation to guide genetic counseling or familial risk assessment.

In this era of precision oncology, the clinical value of germline DDR testing continues to expand. Treatment implications in advanced disease include the FDA-approved indications for olaparib and rucaparib in BRCA-/HRR-mutated mCRPC [24,25] and pembrolizumab in MSI-high/TMB-high metastatic PCa [26]. More recent clinical trials are investigating earlier-stage application and combination approaches for PARP inhibition [27]. While our study did not evaluate treatment outcomes directly, the observed distribution of germline DDR alterations overlaps with patterns described in prior studies, although direct clinical or therapeutic inferences cannot be drawn from the present dataset. In this context, NCCN guidelines also currently recommend germline testing in the setting of metastatic, high-risk localized and node-positive disease, as well as in those with suggestive family history patterns [4]. Our data are consistent with current guideline-based recommendations supporting germline testing in these clinical contexts.

Notably, all pathogenic and likely pathogenic germline variants identified in this cohort were present in the heterozygous state. This observation is consistent with prior germline DDR studies in prostate cancer, in which monoallelic alterations predominate. Importantly, accumulating evidence indicates that heterozygous loss of DDR genes may be biologically relevant through mechanisms such as haploinsufficiency, leading to impaired DNA repair capacity and genomic instability even in the absence of biallelic inactivation [28].

Beyond variant zygosity, the functional consequences of truncating germline DDR alterations warrant careful consideration. While truncating mutations are generally considered to result in loss of function, accumulating experimental evidence suggests that their biological impact may be context-dependent. In DDR pathways characterized by functional redundancy and network-level compensation, the deleterious effects of certain truncating mutations may be partially mitigated through compensatory over-expression or activation of other DNA repair genes [29]. Recent studies have demonstrated that loss-of-function alterations in specific DDR components can be functionally suppressed by enhanced activity of parallel repair pathways, thereby modulating genomic instability and cellular fitness [30,31]. Accordingly, truncating germline DDR variants should not be assumed to confer uniform biological or clinical consequences, further underscoring the need for cautious interpretation of individual variants in the absence of functional validation.

The absence of integrated somatic sequencing and treatment outcome data limits our ability to draw conclusions regarding treatment response or prognostic impact. Several limitations merit consideration. First, the retrospective design introduces the possibility of selection bias, particularly regarding which patients were referred for testing and the completeness of accompanying clinical records. Second, although the sample size is comparable to similar real-world genetic studies, the study may have been underpowered to detect more subtle clinicopathologic associations. Third, long-term oncologic outcomes were not consistently available, limiting our ability to correlate DDR status with survival endpoints. Nonetheless, the strengths of this study include the use of a broad multi-gene panel, rigorous ACMG-based variant interpretation, and representation of a population rarely included in genomic analyses.

In summary, this study provides a detailed characterization of germline DDR alterations in a real-world cohort of Turkish men with prostate cancer. By integrating structured clinicopathological data with ACMG-guided variant interpretation, our findings add population-specific insight to the existing literature. Importantly, these observations are insufficient to support germline-based risk stratification in clinical practice.

Overall, this work should be viewed as complementary and hypothesis-generating, contributing to the broader understanding of germline DDR variation in prostate cancer rather than defining immediate changes to clinical practice.

## 5. Conclusions

Germline DDR variants—most notably BRCA2, CHEK2 and ATM—occur in a relevant subset of PCa patients and tend to associate with higher-grade disease, although without significant differences in age or metastatic presentation. The high prevalence of VUS underlines the interpretive challenges inherent to multigene testing, particularly in populations with limited representation in reference databases.

Overall, this study adds population-specific insight into the landscape of germline DDR variation in prostate cancer and should be viewed as complementary and hypothesis-generating. Larger, integrative studies incorporating both germline and somatic sequencing, as well as longitudinal clinical outcomes, will be required to clarify genotype–phenotype relationships and define the clinical relevance of individual DDR alterations.

## Figures and Tables

**Table 1:** Distribution of germline variant categories according to ISUP Grade Group.

ISUP Risk Groups	No Mutations	Pathogenic/ Likely Pathogenic	VUS	Undefined	Total
ISUP 1–2	18 (81.85%)	1 (4.5%)	3 (13.6%)	0 (0%)	22
ISUP 3	16 (66.7%)	3 (12.5%)	5 (20.8%)	0 (0%)	24
ISUP 4–5	51 (67.1%)	8 (10.5%)	16 (21.1%)	1 (1.3%)	76
Total	85 (69.7%)	12 (9.8%)	24 (19.7%)	1 (1.3%)	122

## Data Availability

The datasets generated and analyzed during the current study are available from the corresponding author upon reasonable request. All variant classifications are provided within the manuscript and Supplementary Materials.

## Supplementary Materials

The following supporting information can be downloaded at: https://www.mdpi.com/article/10.3390/genes17010023/s1, Table S1: Individual clinical and germline genetic characteristics of the study cohort (*n* = 122).
