# Supplementary material for: Prevalence and Clinical Associations of Germline DDR Variants in Prostate Cancer: Real-World Evidence from a 122-Patient Turkish Cohort
Source: Genes (Basel). 2025 Dec 26;17(1):23. doi: 10.3390/genes17010023 (PMC12841403; doi:10.3390/genes17010023)
Supplement: Supplementary file 1 [file genes-17-00023-s001.zip › genes-4057373-supplementary.pdf]

Case Number	Age at diagnose	ISUP Grade Group	Metastasis site	Genetic variation	ClinVarID	Interpretation
1	65.25	1	Unavailable	MUTYH(NM_001048174.2):c.32G>A(p.Gly11Asp) Heterozygous	41763	VUS (Likely Benign)
2	65.91	3	Unavailable	No variant detected		
3	77.43	4	Unavailable	RB(NM_000321.3):c.2626C>T(p.Arg876Cys) Heterozygous	92842	VUS
4	66.03	4	Unavailable	No variant detected		
5	58.86	5	Unavailable	STK11(NM_000455.5):c.362A>T(p.Glu121Val) Heterozygous	No data available	Novel<br>VUS (PM1, PM2, PP3)
6	66.27	5	Unavailable	MUTYH(NM_001128425.2):c.734G>A(p.Arg245His) Heterozygous	140877	Pathogenic/likely pathogenic
7	55.00	5	Unavailable	CHEK2(NM_007194.4):c.1427C>T(p.Thr476Met) Heterozygous	128060	Likely pathogenic
8	48.00	5	Unavailable	BRCA1(NM_007294.4):c.1082C>G(p.Ser361Ter) Heterozygous	54121	Pathogenic
9	67.35	5	Unavailable	APC(NM_000038.6):c.8402G>A(p.Arg2801Gln) Heterozygous	245699	VUS
10	71.88	5	Unavailable	No variant detected		
11	54.14	1	M0	APC(NM_000038.6):c.4918C>T(p.Arg1640Trp) Heterozygous	142246	VUS
12	44.90	1	M0	No variant detected		
13	68.11	1	M0	No variant detected		
14	57.75	1	M0	No variant detected		
15	71.33	1	M0	No variant detected		
16	60.56	1	M0	No variant detected		
17	68.75	1	M0	No variant detected		
18	73.43	1	M0	No variant detected		
19	60.92	2	M0	CHEK2(NM_007194.4):c.1427C>T(p.Thr476Met) Heterozygous	128060	Likely pathogenic

20	68.72	2	M0	BARD1(NM_000465.4):c.1718T>C(p.Ile573Thr) Heterozygous	127722	VUS
21	55.75	2	M0	No variant detected		
22	69.94	2	M0	No variant detected		
23	60.71	2	M0	No variant detected		
24	54.47	2	M0	No variant detected		
25	62.84	2	M0	No variant detected		
26	63.74	2	M0	No variant detected		
27	65.52	2	M0	No variant detected		
28	64.27	2	M0	No variant detected		
29	71.49	2	M0	No variant detected		
30	81.13	3	M0	RAD50(NM_005732.4):c.326_329del(p.Thr109fs) Heterozygous	128017	Pathogenic
31	53.43	3	M0	MSH6(NM_000179.3):c.1628A>C(p.Lys543Thr) Heterozygous	848505	VUS
32	54.40	3	M0	BRCA1(NM_007294.4):c.887G>C(p.Arg296Thr) Heterozygous / BRCA2(NM_000059.4):c.5590G>A(p.Asp1864Asn) Heterozygous / APC(NM_000038.6):c.1302C>G(p.Asp434Glu) Heterozygous	2017000 141155 1381100	VUS VUS VUS
33	61.29	3	M0	No variant detected		
34	71.66	3	M0	No variant detected		
35	59.26	3	M0	No variant detected		
36	66.46	3	M0	No variant detected		
37	69.83	3	M0	No variant detected		
38	51.19	3	M0	No variant detected		
39	63.21	3	M0	No variant detected		
40	54.21	4	M0	CHEK2(NM_007194.4):c.350G>C(p.Arg117Thr) Heterozygous	1732149	VUS

41	70.55	4	M0	CHEK2(NM_007194.4):c.1427C>T(p.Thr476Met) Heterozygous	128060	Likely pathogenic
42	63.44	4	M0	BRCA2(NM_000059.4):c.3751dup(p.Thr1251fs) Heterozygous	51516	Pathogenic
43	54.13	4	M0	No variant detected		
44	54.89	4	M0	No variant detected		
45	66.94	5	M0	NBN(NM_002485.5):c.657_661del(p.Lys219fs) Heterozygous	6940	Pathogenic
46	62.85	5	M0	No variant detected		
47	61.81	5	M0	No variant detected		
48	63.63	5	M0	No variant detected		
49	81.13	5	M0	No variant detected		
50	63.49	3	Lymph Node metastasis	No variant detected		
51	69.26	3	Lymph Node metastasis	No variant detected		
52	56.78	4	Lymph Node metastasis	No variant detected		
53	62.48	4	Lymph Node metastasis	No variant detected		
54	79.43	5	Lymph Node metastasis	RB1(NM_000321.3):c.2587G>A(p.Ala863Thr) Heterozygous	960039	VUS
55	35.33	5	Lymph Node metastasis	No variant detected		
56	62.86	5	Lymph Node metastasis	No variant detected		
57	77,51	5	Lymph Node metastasis	No variant detected		
58	67.09	5	Lymph Node metastasis	No variant detected		
59	63.78	2	Bone metastasis	No variant detected		

60	63.72	2	Bone metastasis	No variant detected		
61	58.04	3	Bone metastasis	MRE11(NM_005591.3):c.1462C>T(p.Arg488Cys) Heterozygous	127972	VUS
62	74.29	3	Bone metastasis	BRIP1(NM_032043.2):c.653G>A(p.Cys218Tyr) Heterozygous	407824	VUS
63	75.78	3	Bone metastasis	ATM(NM_000051.4):c.3576G>A(p.Lys1192=) Heterozygous	3035	Pathogenic
64	60.90	3	Bone metastasis	No variant detected		
65	72.37	3	Bone metastasis	No variant detected		
66	60.90	3	Bone metastasis	No variant detected		
67	52.48	3	Bone metastasis	No variant detected		
68	54.88	3	Bone metastasis	No variant detected		
69	62.94	3	Bone metastasis	No variant detected		
70	64.98	4	Bone metastasis	POLD1(NM_002691.4):c.14G>A(p.Arg5Gln) Heterozygous	408051	VUS
71	74.26	4	Bone metastasis	NF1(NM_001042492.3):c.1921A>G(p.Ser641Gly) Heterozygous	231483	VUS
72	71.89	4	Bone metastasis	ATM(NM_000051.4):c.6671T>C(p.Met2224Thr) Heterozygous	181884	VUS
73	57.39	4	Bone metastasis	BRCA1(NM_007294.4):c.4472C>G(p.Pro1491Arg) Heterozygous	953812	VUS
74	71.25	4	Bone metastasis	No variant detected		
75	72.10	4	Bone metastasis	No variant detected		
76	76.77	4	Bone metastasis	No variant detected		

77	68.34	4	Bone metastasis	No variant detected		
78	62.17	4	Bone metastasis	No variant detected		
79	69.27	4	Bone metastasis	No variant detected		
80	68.01	4	Bone metastasis	No variant detected		
81	65.39	4	Bone metastasis	No variant detected		
82	66.64	4	Bone metastasis	No variant detected		
83	65.55	4	Bone metastasis	No variant detected		
84	51.23	5	Bone metastasis	PMS2(ENST00000265849.12):c.2405G>A(p.Arg802Gln) Heterozygous	411071	VUS
85	68.24	5	Bone metastasis	MLH1(NM_000249.4):c.545+6G>C Heterozygous	630938	VUS
86	66.39	5	Bone metastasis	CHEK2(NM_007194.4):c.1091T>C(p.Ile364Thr) Heterozygous<br>RAD51C(NM_058216.3):c.14C>T(p.Thr5Met) Heterozygous	185975<br>128203	VUS<br>VUS
87	61.84	5	Bone metastasis	BRCA2(NM_000059.4):c.7006C>T(p.Arg2336Cys) Heterozygous	96845	VUS
88	75.21	5	Bone metastasis	BRCA2(NM_000059.4):c.4159T>A(p.Leu1387Ile) Heterozygous	96804	VUS
89	82.06	5	Bone metastasis	BRCA1(NM_007294.4):c.1745C>T(p.Thr582Met) Heterozygous	185698	VUS
90	64.97	5	Bone metastasis	ATM(NM_000051.4):c.6154G>A(p.Glu2052Lys) Heterozygous	181975	* Conflicting classifications of pathogenicity
91	59.92	5	Bone metastasis	ATM(NM_000051.4):c.1564_1565del(p.Glu522fs) Heterozygous	127340	Pathogenic
92	55.06	5	Bone metastasis	No variant detected		

93	48.36	5	Bone metastasis	No variant detected		
94	59.42	5	Bone metastasis	No variant detected		
95	57.22	5	Bone metastasis	No variant detected		
96	75.27	5	Bone metastasis	No variant detected		
97	46.99	5	Bone metastasis	No variant detected		
98	47.01	5	Bone metastasis	No variant detected		
99	54.98	5	Bone metastasis	No variant detected		
100	80.72	5	Bone metastasis	No variant detected		
101	59.51	5	Bone metastasis	No variant detected		
102	48.33	5	Bone metastasis	No variant detected		
103	67.73	5	Bone metastasis	No variant detected		
104	77.70	5	Bone metastasis	No variant detected		
105	74.30	5	Bone metastasis	No variant detected		
106	55.36	5	Bone metastasis	No variant detected		
107	79.84	5	Bone metastasis	No variant detected		
108	55.36	5	Bone metastasis	No variant detected		
109	65.15	5	Bone metastasis	No variant detected		

110	64.20	5	Bone metastasis	No variant detected		
111	64.52	5	Bone metastasis	No variant detected		
112	80.75	5	Bone metastasis	No variant detected		
113	71.95	3	Visceral metastasis	STK11(NM_000455.4):c.1150C>T(p.Arg384Trp) Heterozygous	234419	VUS
114	66.54	3	Visceral metastasis	CHEK2(NM_007194.4):c.1100del(p.Thr367fs) Heterozygous	128042	Pathogenic
115	68.90	4	Visceral metastasis	BRIP1(NM_032043.3):c.1945_1950del(p.Gly649_Thr650del) Heterozygous<br><br>BRCA1(NM_007294.4):c.2525A>C(p.Glu842Ala) Heterozygous	No data available<br><br>No data available	** Novel<br>VUS (PM2, PM4)<br>** Novel<br>VUS (PM2)
116	68.18	4	Visceral metastasis	No variant detected		
117	67.42	4	Visceral metastasis	No variant detected		
118	74.43	4	Visceral metastasis	No variant detected		
119	70.27	5	Visceral metastasis	MSH3(NM_002439.5):c.3046_3050delinsTCA(p.Glu1016SerfsTer20) Heterozygous	No data available	*** Novel<br>Likely pathogenic (PVS1, PM2)
120	69.79	5	Visceral metastasis	No variant detected		
121	72.76	5	Visceral metastasis	No variant detected		
122	73.34	5	Visceral metastasis	No variant detected		
Total	122	122	112	122	122	122

Table-S1: Individual clinical and germline genetic characteristics of the study cohort (n=122).

Metastasis status was unavailable for the first ten patients due to incomplete historical clinical records at the time of diagnosis. *M0* indicates nonmetastatic disease.

*Case 90: This variant shows conflicting classifications in ClinVar submissions (pathogenic, likely pathogenic, and uncertain significance).

**Case 115: This variation has not yet been deposited in the ClinVar database; however, it was classified as a variant of uncertain significance (VUS) based on concordant interpretations in the VarSome and Franklin databases, in accordance with ACMG/AMP variant interpretation criteria.
